# Outcomes of Brentuximab Vedotin Monotherapy in Refractory/Relapsed Classical Hodgkin’s Lymphoma: A Multi-Center Retrospective Study on Survival and Safety

**DOI:** 10.1007/s12288-025-02093-w

**Published:** 2025-07-16

**Authors:** Mehmet Bakirtas, Ipek Yonal Hindilerden, Bahar Uncu Ulu, Omer Ekinci, Mehmet Hilmi Dogu, Ismet Aydogdu, Ilhami Berber, Duzgun Ozatli, Sinan Demircioglu, Gulsum Akgun Cagliyan, Sinem Namdaroglu, Tarik Onur Tiryaki, Tugce Nur Yigenoglu, Istemi Serin, Burhan Turgut, Murat Albayrak, Yavuz Katircilar, Tuba Hacıbekiroglu, Meliha Nalcaci, Mehmet Ali Erkurt, Mehmet Sinan Dal, Serdal Korkmaz, Turgay Ulas, Fevzi Altuntas

**Affiliations:** 1 Department of Hematology, Tekirdag Dr. Ismail Fehmi Cumalioglu City Hospital, Istiklal Mahallesi, Yemen Sokak No: 13, Süleymanpaşa, Tekirdağ, 59030 Turkey; 2 Department of Internal Medicine, Division of Hematology, Istanbul University Istanbul Medical Faculty, Istanbul, Turkey; 3 Department of Hematology & Apheresis Unit, University of Health Sciences, Ankara Oncology Training and Research Hospital, Ankara, Turkey; 4 Şişli Kolan International Hospital Istanbul, Hematology and Bone Marrow Transplantation Unit, Istanbul, Turkey; 5 Depatment of Hematology, Istinye University, Istanbul, Turkey; 6 Faculty of Medicine, Department of Hematology, Celal Bayar University, Manisa, Turkey; 7 Faculty of Medicine, Department of Hematology, Inonu University, Malatya, Turkey; 8 Faculty of Medicine, Department of Hematology, Ondokuz Mayis University, Samsun, Turkey; 9 Meram Faculty of Medicine, Department of Hematology, Necmettin Erbakan University, Konya, Turkey; 10 Faculty of Medicine, Department of Hematology, Pamukkale University, Denizli, Turkey; 11 Faculty of Medicine, Department of Hematology, Dokuz Eylul University, Izmir, Turkey; 12 Department of Hematology, Agri Training and Research Hospital, Agri, Turkey; 13 Faculty of Medicine, Department of Hematology, Tekirdag Namik Kemal University, Tekirdag, Turkey; 14 Department of Hematology, University of Health Sciences, Diskapi Yildirim Beyazit Training and Research Hospital, Ankara, Turkey; 15 Kayseri Medical Faculty, Department of Hematology and Stem Cell Transplantation Unit, University of Health Sciences, Kayseri, Turkey; 16 Faculty of Medicine, Department of Hematology, Sakarya University, Sakarya, Turkey; 17 School of Medicine, Department of Internal Medicine, Division of Hematology, Ankara Yildirim Beyazit University, Ankara, Turkey

**Keywords:** Hodgkin’s lymphoma, Brentuximab vedotin, Hematopoietic stem cell transplantation, Survival analysis, Adverse events

## Abstract

**Background:**

Refractory/relapsed classical Hodgkin’s lymphoma (R/RcHL) poses significant treatment challenges, with limited success rates in achieving long-term remission. Brentuximab Vedotin (BV), an anti-CD30 monoclonal antibody-drug conjugate, has emerged as a promising therapeutic option. This study aims to evaluate the efficacy and safety of BV monotherapy in R/RcHL patients, particularly regarding survival outcomes and adverse events.

**Methods:**

This multi-center retrospective study included 82 R/RcHL patients aged 18 and over, treated with BV monotherapy across 14 institutions in Turkey from June 2012 to June 2020. Data on demographics, clinical characteristics, treatment response, adverse events, and overall survival (OS) rates were collected and analyzed. Primary outcomes were overall treatment response rate and OS, while the secondary outcome focused on the adverse event profile of BV treatment.

**Results:**

Among the patients (56.1% female, median age 33.5 years), the overall treatment response rate was 76.8%. The median OS was 13.6 months, with patients undergoing hematopoietic stem cell transplantation (HSCT) post-BV treatment exhibiting significantly longer survival (19.6 months) compared to those who did not receive HSCT (7.8 months, *p* < 0.001). Grade 3 to 5 adverse events were observed in 32.9% of patients, with neutropenia being the most common.

**Conclusion:**

BV monotherapy demonstrates substantial efficacy in treating R/RcHL, offering a favorable balance between treatment response and manageable adverse events. Particularly effective as a bridging therapy to HSCT, BV significantly extends survival in R/RcHL patients. These findings underscore the need for prospective studies to further delineate patient subsets most likely to benefit from BV monotherapy.

**Supplementary Information:**

The online version contains supplementary material available at 10.1007/s12288-025-02093-w.

## Introduction

Patients with refractory/relapsed classical Hodgkin’s lymphoma (R/RcHL) commonly have a poor prognosis [1, 2]. Approximately 20–30% of cHL patients experience either relapse or resistance to first-line treatment [3, 4]. Although autologous hematopoietic stem cell transplantation (AHSCT) is the standard treatment for patients with R/RcHL, only half of R/RcHL patients who undergo AHSCT survive [2, 3]. Various agents, including BV, have been developed, and their use as salvage therapy or maintenance therapy after AHSCT has improved the outcomes of R/R HL patients undergoing AHSCT [3, 5, 6]. The use of BV, a conjugate of an anti-CD30 (cluster of differentiation 30) monoclonal antibody and monomethyl auristatin E, is increasing as a first-line treatment for advanced disease, as part of salvage therapy before AHSCT, and as maintenance therapy after AHSCT [3]. Previous studies have reported up to 92% overall response rate (ORR)to BV monotherapy in R/RcHL patients, depending on patient and treatment characteristics [7–12]. Since the optimal treatment of R/RcHL disease has not been fully established and the complete remission (CR) rate is less than 40%, the use of BV monotherapy, even in combination with other drugs, has been investigated to achieve more promising results [4, 5, 13–16]. Additionally, long-term use of BV has been associated with the development of hematological and non-hematological toxicities. As a matter of fact, the development of such toxicities often leads to early discontinuation of BV treatment [13–17]. All in all, real-world evidence regarding the activity and tolerability of BV monotherapy in R/RcHL remains insufficient [7, 12, 17–19]. Therefore, the objective of this study is to evaluate survival and safety outcomes in R/RcHL patients who received BV as the salvage monotherapy before and/or after AHSCT/allogeneic hematopoietic stem cell transplantation (alloHSCT).

## Materials and Methods

### Study Design

This study was designed as a retrospective multi-center study, including 14 institutions across Turkey. The study protocol was approved by the Medical Sciences University Dr Abdurrahman Yurtaslan Ankara Oncology Education and Research Hospital ethical committee (Approval Date:28.07.2021, Approval No: 2021-07/ 1309). The study was conducted in accordance with the ethical principles set forth in the Declaration of Helsinki. Given the study’s retrospective design and the collected data’s unanimity, written informed consent was not obtained from the patients included in the study.

### Population and Sample

The study population consisted of all consecutive patients with R/RcHL aged 18 and older who received BV monotherapy as the salvage therapy to treat or control the disease between June 2012 and June 2020. Patients with classical Hodgkin’s lymphoma (cHL) who either failed to achieve complete remission (CR) after conventional first-line treatment (primary refractory), or relapsed at any time following an initial response, were considered to have relapsed/refractory (R/R) cHL. This included patients who received BV as salvage therapy after undergoing AHSCT or alloHSCT, and patients ineligible for transplantation who had received at least two lines of multi-agent chemotherapy [16, 18]. BV-naive patients, patients who received BV salvage therapy in combination with other treatments, patients with an autoimmune disease, and patients with an Eastern Cooperative Oncology Group Performance Status (ECOG-PS) of 3 or 4 were excluded from the study. This study focused on BV monotherapy to evaluate its efficacy and safety as a single-agent treatment in real-world settings. BV was chosen as a single-agent therapy for patients with poor performance status, prior toxicity to multi-agent chemotherapy, or in cases where clinical judgment deemed it most appropriate. In the end, 82 patients treated with BV salvage monotherapy for R/RcHL were included in the study sample.

### BV Treatment

All patients received an outpatient infusion of 1.8 mg/kg BV for 30 min every three weeks. Treatment duration was individualized based on treatment response, treatment tolerability, consolidation strategies, and availability of alternative treatments [18]. The response of the tumor to BV monotherapy was assessed based on the International Working Group Revised Response criteria [18]. Safety and tolerability of BV were evaluated according to the National Cancer Institute Common Terminology Criteria for Adverse Events Version 4.0 [20]. BV dose was reduced to 1.2 mg/kg in patients who developed grade 3 toxicity due to BV.

### Data Collection

Patients’ demographic (age, gender), clinical (age at diagnosis, B symptoms, disease stage, histological subtypes, bulky tumor), laboratory (hemoglobin, lactate dehydrogenase, and albumin levels, leukocyte, absolute lymphocyte, and thrombocyte counts, and sedimentation rate), treatment (the conventional salvage chemotherapy regimens, AHSCT or alloHSCT received, and the responses of the tumor to these treatments) characteristics were obtained from their medical files, and recorded. BV treatment data were also noted, including the time, dose, number, and duration of treatments, treatment response, and adverse events. In addition, patient follow-up data, including survival outcomes, disease progression after BV, and reasons for discontinuing BV, were recorded. Response to BV treatment was assessed using PET-CT or CECT, depending on institutional protocols. Reassessments were conducted after every three treatment cycles or as clinically indicated. The International Working Group Revised Response Criteria were used to classify treatment outcomes. The study’s primary outcomes were ORR, which included patients with CR and partial remission (PR) and overall survival (OS) after the initiation of BV treatment [7, 12]and the secondary outcome was the adverse event-based safety profile of BV treatment. OS was defined as the time from the initiation of BV treatment to the last follow-up time or death from any cause [18]. The OS was censored at the date of the last information. Progression-free survival (PFS) was defined as the time from the initiation of BV treatment to the date of documented disease progression or death from any cause. Patients who were alive without disease progression at the last follow-up were censored. The timeline for reassessment scans varied across the participating centers due to differences in institutional protocols and clinical practices. In some institutions, reassessments were conducted at predefined intervals, such as after every three treatment cycles. In other centers, the timing was determined based on clinical judgment or patient-specific factors, including response to treatment or the presence of adverse events. The data were collected from a centralized database maintained by [name of institutions/consortium], which includes detailed records of HL patients. For institutions without centralized records, electronic medical systems were searched using specific keywords (‘Hodgkin lymphoma’, ‘relapse’, ‘refractory’) to identify relevant cases. We also analyzed the OS of the R/RcHL patients with and without AHSCT or alloHSCT after BV treatment.

### Statistical Analysis

The descriptive statistics obtained from the collected data were tabulated as mean ± standard deviation or median with minimum and maximum values in the case of continuous (numerical) variables and as numbers and percentages in the case of categorical variables. The normal distribution characteristics of the numerical variables were analyzed using Shapiro-Wilk, Kolmogorov-Smirnov, and Anderson-Darling tests. Univariate and multivariate linear regression analyses were conducted to assess the effects of each independent demographic and clinical variable on OS following the initiation of BV treatment. Accordingly, in the univariate analysis, the impacts of age at the initiation of BV treatment, gender, ECOG-PS before BV treatment, B symptoms, bulky tumor, number of previously received regimens, AHSCT and alloHSCT therapy, and number of BV treatments on OS were examined separately. An odds ratio (OR), 95% confidence interval (CI), and a *p*-value were calculated for each variable. Subsequently, the variables that were found to have a significant effect on OS in the univariate analyses were further assessed for their impact on OS in the multivariate analysis. An OR, 95% CI, and a *p*-value were calculated for each variable examined within the scope of the multivariate analysis. Statistical analyses were conducted using Jamovi project 2.3.28 (Jamovi, version 2.3.28.0, 2023, retrieved from https://www.jamovi.org) and JASP 0.18.3 (Jeffreys’ Amazing Statistics Program, version 0.18.3, 2024, retrieved from https://jasp-stats.org). Probability (*p*) statistics of ≤ 0.05 were deemed to indicate statistical significance.

## Results

The study sample consisted of 82 patients with R/RcHL, 46 (56.1%) female and 36 (43.9%) male. The median age of the patients was 33.5 (min. 18, max. 85) years. On the other hand, the median age of the patients when they were first diagnosed was 29 years. B symptoms were detected in 68.3% of the patients when they were first diagnosed. Nodular sclerosis (64.6%) and mixed cellularity (25.6%) were the most common histological types. Stage III disease was detected in 38(46.3%) patients, stage IV disease in 25 (30.5%), and stage II disease in 18 (22.0%). Among the 82 patients included in the study: 47 (57.3%) patients did not achieve CR after conventional treatment, 30 (36.6%) experienced relapse within three months, 4 (4.7%) received BV as salvage therapy after AHSCT/alloHSCT, and 47 (57.3%) were ineligible for transplantation and received at least two multi-agent conventional chemotherapy regimens following relapse. The baseline characteristics of the patients when they were first diagnosed are given in Supplementary Material 1.

Most (84.1%) patients had an ECOG-PS score of 0 or (1) B symptoms and bulky tumors were observed in 36 (43.9%) and 10 (12.2%) patients, respectively. The median number of conventional chemotherapy regimens the patients received before the BV treatment was 3 (min. 1, max. 10). Chemotherapy, which was administered to 81 (98.8%) patients, was the most applied treatment modality, followed by AHSCT and alloHSCT, which were administered to 34 (41.5%) and 4 (4.7%) patients, respectively. The rate of patients with a transplant failure before BV treatment was 41.5% (*n* = 34). The clinical characteristics of the patients at the initiation of BV treatment and the combination therapies they received before the BV treatment are detailed in Tables 1 and 2. Prior to BV treatment, 4.7% (*n* = 4) of patients underwent alloHSCT.

**Table 1:** Clinical characteristics of the patients at the initiation of Brentuximab Vedotin treatment

	Overall (*n* = 82)
**Age for BV treatment** ^†^	32.0 [18.0–93.0]
**ECOG-PS before BV treatment** ^‡^	
0	23 (28.0)
1	46 (56.1)
2	13 (15.9)
**ECOG-PS score groups** ^‡^	
0–1	69 (84.1)
2	13 (15.9)
**B-symptoms** ^‡^	36 (43.9)
**Bulky tumor** ^‡^	10 (12.2)
**Number of prior regimens** ^†^	3.0 [1.0–10.0]
**Type of prior treatment modalities** ^‡^	
Chemotherapy	81 (98.8)
AHSCT	34 (41.5)
AlloHSCT	4 (4.7)
**Prior treatment combinations** ^**‡**^	
Only chemotherapy	47 (57.3)
Chemotherapy + AHSCT	30 (36.6)
Chemotherapy + AHSCT + AlloHSCT	4 (4.9)

†: median [min-max], ‡: n (%)

ECOG-PS: the Eastern Cooperative Oncology Group Performance Status, BV: brentuximab vedotin, AHSCT: autologous hematopoietic stem cell transplantation, alloHSCT: allogeneic hematopoietic stem cell transplantation

**Table 2:** Clinical characteristics of the patients at the initiation of Brentuximab Vedotin treatment

	Overall (*n* = 82)
**Number of BV treatment** ^†^	6.0 [2.0–26.0]
**Median time from initial diagnosis to first dose (month)** ^†^	23.6 [0.4–305.3]
Duration of BV treatment (month) ^†^	4.8 [1.0–25.8]
**Dose reduction** ^‡^	6 (7.3)
**ORR (CR + PR)** ^‡^	63 (76.8)
**Response categories** ^‡^	
CR	33 (40.2)
PR	30 (36.6)
PD	17 (20.7)
SD	2 (2.4)

†: median [min-max], ‡: n (%)

BV: brentuximab vedotin, ORR: overall response rate, CR: clinical remission, PR: partial remission PD: progressive disease, SD: stable disease

### Survival Outcomes

The median follow-up duration for the entire study population was 6.0 months (range: 1.0–60.3 months), calculated using the statistical median method. Due to the retrospective design and non-uniform follow-up intervals across centers, the reverse Kaplan-Meier method was not applied. During the follow-up period, 19 patients (23.2%) died. Based on available clinical records, the majority of deaths were attributed to disease progression, while a smaller subset was associated with treatment-related complications, such as infections and organ dysfunction. However, due to limitations in retrospective documentation, the exact cause of death could not be determined for all cases.

**Fig. 1:**
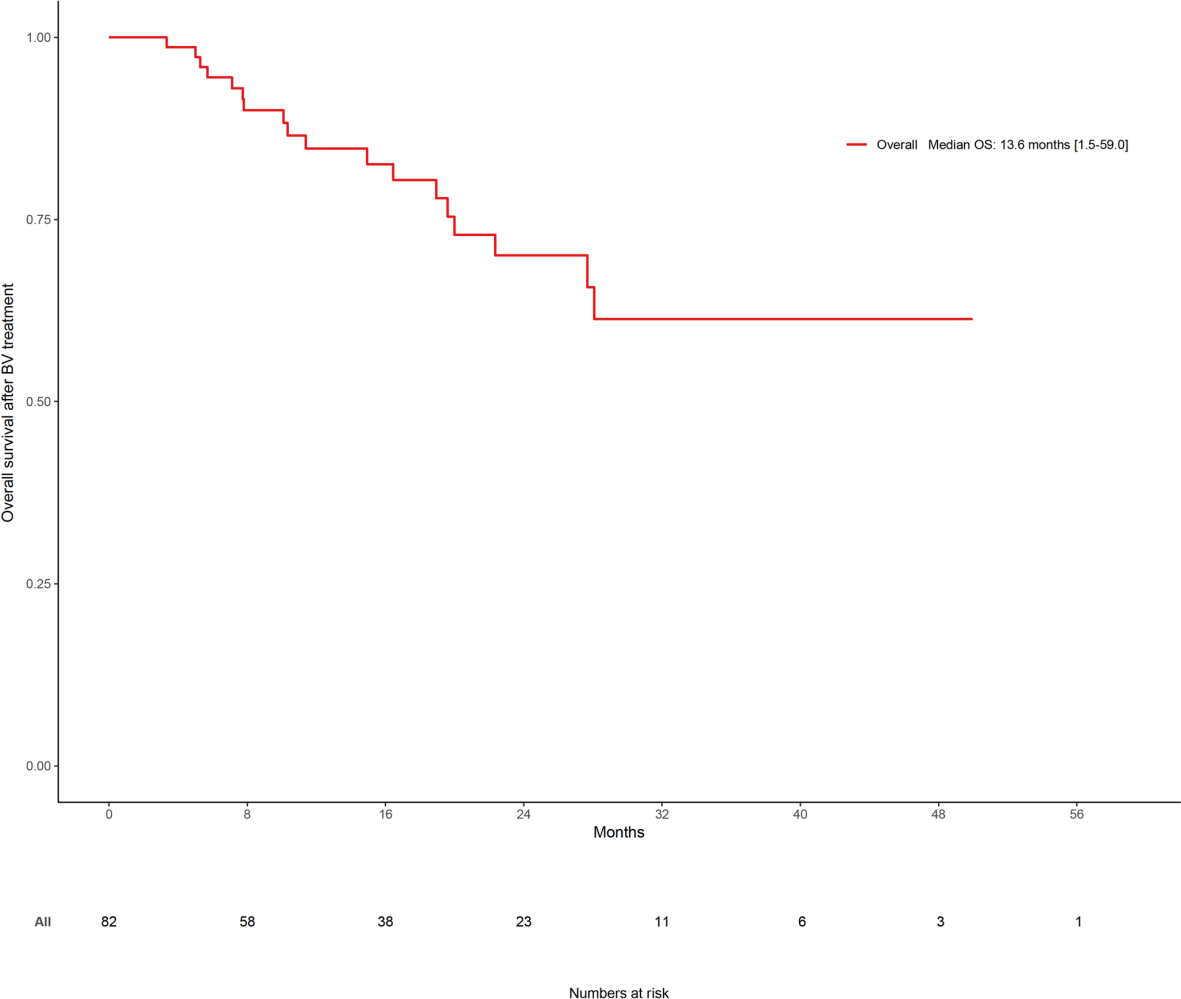
Kaplan-Meier plots of probabilities of overall survival (OS) (months) vs. timeline (months) of the study population (*n* = 82)

**Fig. 2:**
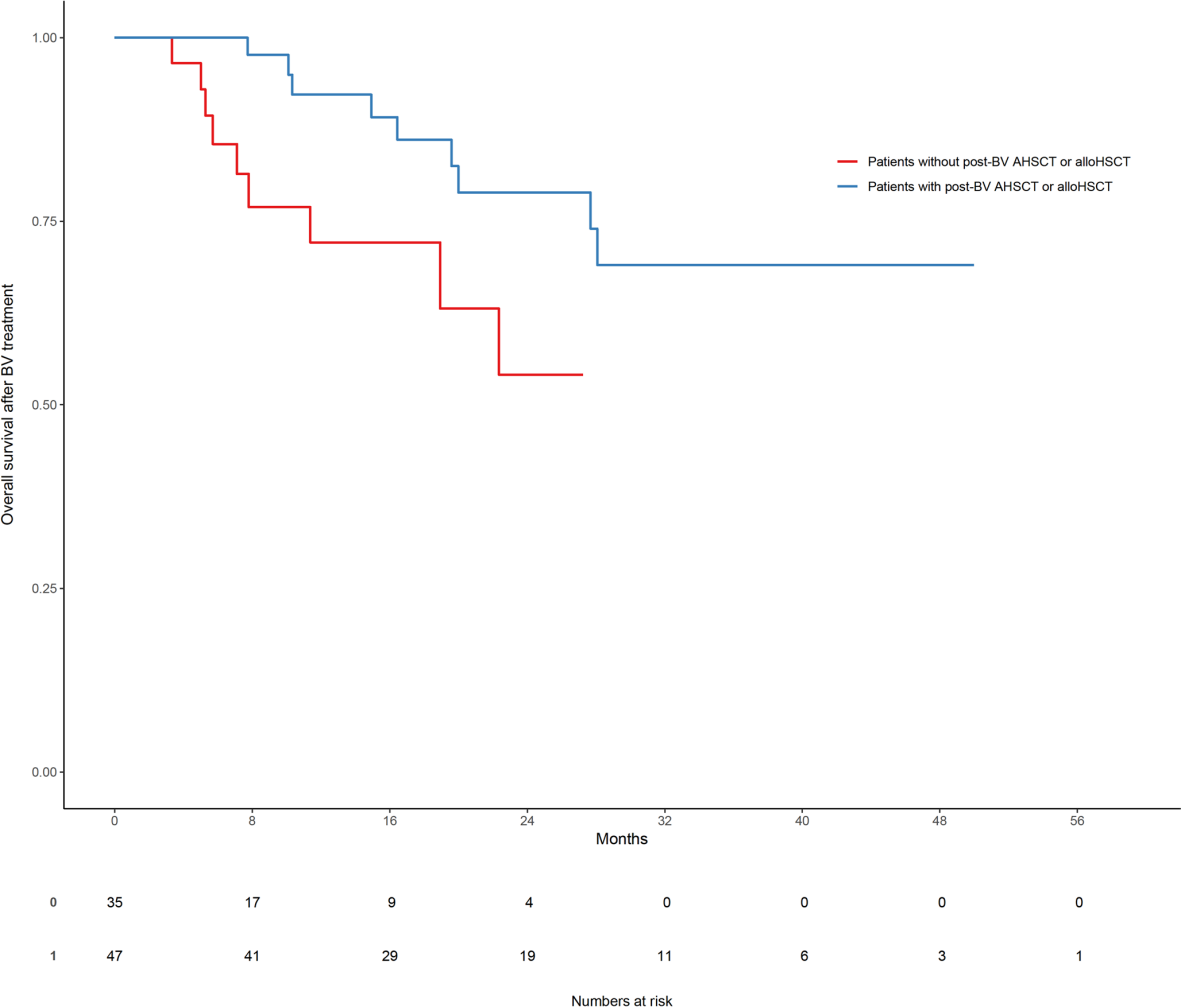
Comparison of the length of survival after initiation of brentuximab vedotin treatment in R/R cHL patients with (blue) and without (red) AHSCT or alloHSCT

The median progression-free survival (PFS) was 6.8 months (range: 1.0–20.2 months), and the median overall survival (OS) following BV initiation was 13.6 months (range: 1.5–59.0 months) (Table 1; Fig. 1). When stratified by post-BV transplantation status, OS was significantly longer in patients who underwent autologous or allogeneic HSCT compared to those who did not. Specifically, the median OS was 19.6 months (range: 2.8–59.0 months) in the HSCT group versus 7.8 months (range: 1.5–27.2 months) in the non-transplanted group (*p* < 0.001, Fig. 2).

### Predictors of Survival

Univariate logistic regression analysis was conducted to identify factors associated with overall survival (OS). Variables assessed included age at BV initiation, sex, ECOG performance status, B symptoms, bulky disease, number of prior treatment regimens, and transplantation status (pre- or post-BV). In univariate analysis, the presence of B symptoms, the number of BV cycles, and post-BV HSCT were found to be associated with OS.

In multivariate analysis, only post-BV autologous or allogeneic HSCT remained significantly associated with improved OS (OR = 7.48; 95% CI: 2.11–12.86; *p* = 0.008) (Table 2). This finding highlights the crucial role of HSCT consolidation in prolonging survival following BV therapy in patients with R/RcHL. Due to data limitations, subgroup analyses for PFS were not feasible. Further prospective studies with standardized follow-up protocols are needed to better assess factors influencing PFS in this population.

The median number of BV treatment cycles patients received was 6 (min.2, max. 26). The other BV-related characteristics are given in Table 3. ORR, CR, and PR were achieved in 63 (76.8%), 33 (40.2%), and 30 (36.6%) patients, respectively. The remaining 17 (20.7%) and 2 (2.4%) patients had progressive disease (PD) and stable disease (SD), respectively. BV treatment was discontinued in 72 (87.8%) patients. The most common reason for the discontinuation of BV was AHSCT (34.1%), followed by alloHSCT (23.2%) and PD (18.3%) (Table 2). The primary reason for discontinuation of BV was consolidation by either autologous or allogeneic HSCT (57.3%).

**Table 3:** Demographic and clinical characteristics of the study group at initial diagnosis

	Overall (*n* = 82)
**Age (year)** ^†^	29.0 [17.0–85.0]
**ECOG-PS score** ^‡^	
0	22 (26.8)
1	49 (59.8)
2	11 (13.4)
**B symptoms** ^‡^	56 (68.3)
**Histology** ^‡^	
Nodular sclerosis	53 (64.6)
Mixed cellularity	21 (25.6)
Lymphocyte-rich	6 (7.3)
Lymphocyte-depleted	2 (2.4)
**Disease stage** ^‡^	
1	1 (1.2)
2	18 (22.0)
3	38 (46.3)
4	25 (30.5)
**Extranodular involvement** ^‡^	23 (28.0)
**Bulky tumor** ^‡^	19 (23.2)
**Bone marrow involvement** ^‡^	14 (17.1)

†: median [min-max], ‡: n (%)

ECOG-PS: the Eastern Cooperative Oncology Group Performance Status

There were 119 adverse events in 82 patients. Twenty-eight (32.9%) patients experienced grade 3 to 5 adverse events. Neutropenia grade 3 to 5 was the most common adverse event type, seen in 24 (29.3%) patients, followed by neuropathy (6.1%) and pneumonia grade 3 to 5 (6.1%). The details of the adverse events observed in the study group are given in Tables 4 and 5.

**Table 4:** Treatment outcomes and survival data

	Overall (*n* = 82)
**Length of follow up after BV treatment (month)** ^†^	6.0 [1.0–60.3]
**Post-BV progression** ^‡^	17 (20.7)
**Patients with end of treatment** ^‡^	72 (87.8)
**Reason for end of treatment** ^‡^	
AHSCT	28 (34.1)
AlloHSCT	19 (23.2)
Progression	15 (18.3)
Side effects	4 (4.9)
Mortality	6 (7.3)
**Survival after BV treatment**	13.6 [1.5–59.0]
**Outcome** ^‡^	
Survived	63 (76.8)
Nonsurvived	19 (23.2)

†: median [min-max], ‡: n (%)

BV: brentuximab vedotin, AHSCT: autologous hematopoietic stem cell transplantation, alloHSCT: allogeneic hematopoietic stem cell transplantation

**Table 5:** Regression analysis of demographic and clinical characteristics of the patients impacting overall survival after the initiation of Brentuximab Vedotin treatment

	Univariate linear regression		Multivariate linear regression	
	Beta Coefficient [CI 95%]	*p*	Beta Coefficient [CI 95%]	*p*
**Age for BV treatment**	-0.25 [-0.38 – -0.11]	**< 0.001**	-0.12 [-0.25–0.02]	0.087
**Gender**: *Male vs. female*	-0.46 [-6.17–5.24]	0.873	-	-
**ECOG-PS groups before BV treatment**: *2 vs. 0–1*	-8.91 [-16.41 – -1.41]	**0.022**	-2.92 [-9.4–3.56]	0.38
**B symptoms**: *Yes vs. No*	-8.18 [-13.59 – -2.76]	**0.004**	-2.82 [-7.86–2.21]	0.275
**Bulky tumor**: *Yes vs. No*	-3.67 [-12.28–4.94]	0.406	-	-
**Number of prior regimens**: *≥3 vs. <3*	7.36 [1.83–12.9]	**0.011**	4.09 [-0.71–8.89]	0.099
**Prior AHSCT + alloHSCT**: *yes vs. no*	8.66 [3.23–14.08]	**0.002**	5.36 [0.09–10.64]	**0.050**
**Number of BV treatment**	0.88 [0.41–1.35]	**< 0.001**	0.44 [-0.02–0.89]	0.062
**Post-BV AHSCT + alloHSCT**: *yes vs. no*	10.93 [5.74–16.13]	**< 0.001**	7.48 [2.11–12.86]	**0.008**

CI: Confidence interval, BV: brentuximab vedotin, ECOG-PS: the Eastern Cooperative Oncology Group Performance Status, AHSCT: autologous hematopoietic stem cell transplantation, alloHSCT: allogeneic hematopoietic stem cell transplantation

## Discussion

The BV treatment resulted in an ORR of 76.8% in patients with R/RcHL. In addition, approximately two-thirds of the patients with R/RcHL survived after BV treatment. Grade 3 to 5 adverse events were seen in roughly one-third of the patients. These findings are preliminary evidence for BV’s efficacy and safety in treating R/RcHL. The regression analysis provided significant insights into factors influencing overall survival (OS) in patients treated with BV. The univariate analysis identified several predictors of better OS, including post-BV AHSCT or alloHSCT, fewer B symptoms, and a higher number of BV cycles. However, in the multivariate analysis, only post-BV AHSCT/alloHSCT (*p* = 0.008) remained significantly associated with better OS. These findings highlight the pivotal role of consolidative transplantation following BV treatment. Patients who undergo transplantation after BV benefit from enhanced OS, suggesting that BV serves as an effective bridging therapy to transplantation. This emphasizes the importance of timely identification of candidates for transplantation and early initiation of BV to maximize therapeutic benefits. However, large-scale studies with more extended follow-up periods are needed to consider the findings regarding BV’s effectiveness and safety in treating R/RcHL as definitive evidence.

Previous studies have reported up to 92% overall response rate (ORR) to BV monotherapy in R/RcHL patients [7–12]. In a systematic review and meta-analysis, Plattel et al. reported the ORR and CR rates in R/RcHL patients receiving BV monotherapy as 62.6% and 32.9%, respectively [17]. Factors affecting the response to BV treatment include various factors such as patient and disease characteristics, number of previously received treatments, and length of follow-up period. The results of the studies on the characteristics of the subset of patients who could potentially be treated with BV monotherapy are contradictory [7–12, 21, 22].

In one of these studies, Perrot et al. [9] reported that the best response to BV treatment was achieved after administering a median of four cycles. They also mentioned that the response rate decreased from 60.5 to 33.5% at the end of BV treatment, with a median response duration of 8.4 months. Four cycles of BV therapy have been considered optimal for achieving good treatment response with a potential consolidation with transplantation [11]. In comparison, we did not evaluate ORR in this study according to the number of BV cycles administered. Nevertheless, all relevant findings suggest that BV’s efficacy in treating R/RcHL is time-dependent. Therefore, the decision to proceed with AHSCT or alloHSCT must be made in a timely manner to consolidate treatment response or achieve a cure.

The severity and type of adverse effects associated with BV should be considered while adjusting the dosage of BV treatment. In a pivotal study, Chen et al. [23] reported peripheral neuropathy as the most frequent adverse effect seen in patients treated with BV. Neuropathy was observed as a non-hematological toxicity in 6.1% of patients receiving BV treatment in our cohort (Table 6). This incidence aligns with previously reported studies, where peripheral neuropathy was identified as one of the most common adverse effects associated with BV. Neuropathy, particularly in grade 3–5 severity, may necessitate dose reduction or treatment discontinuation. These findings underscore the importance of regular monitoring for neuropathy and the early implementation of supportive care strategies to mitigate its impact on treatment adherence and outcomes. Others reported similar findings [8, 9, 24]. Plattel et al. [17] reported hematological toxicities, including neutropenia, anemia, and thrombocytopenia, as the most common adverse events associated with BV treatment. The relatively higher rates of hematological toxicities were observed in cHL patients who were treated with the combinations of BV [25]. In parallel, we found neutropenia to be the most common adverse effect in our study group.

**Table 6:** Distribution of adverse side events related to Brentuximab Vedotin treatment

	Overall (*n* = 82)
**Neutropenia grade** ^‡^	
0	43 (52.4)
1	11 (13.4)
2	4 (4.9)
3	9 (11.0)
4	13 (15.9)
5	2 (2.4)
**Neutropenia grade 3–5** ^‡^	24 (29.3)
**Pneumonia grade** ^‡^	
0	72 (87.8)
1	2 (2.4)
2	3 (3.7)
3	1 (1.2)
4	3 (3.7)
5	1 (1.2)
**Pneumonia grade 3–5** ^‡^	5 (6.1)
**Neuropathy grade** ^‡^	
0	72 (87.8)
1	4 (4.9)
2	1 (1.2)
3	4 (4.9)
4	1 (1.2)
**Neuropathy grade 3–5** ^‡^	5 (6.1)
**Hyperkalemia grade** ^‡^	82 (100.0)
**Hyperkalemia grade 3–5** ^‡^	0 (0.0)
**Acute renal failure** ^‡^	
0	79 (96.3)
1	2 (2.4)
2	1 (1.2)
**Acute renal failure grade 3–5** ^‡^	0 (0.0)
**Thrombocytopenia grade** ^‡^	
0	54 (65.9)
1	13 (15.9)
2	3 (3.7)
3	4 (4.9)
4	8 (9.8)
**Thrombocytopenia grade 3–5** ^‡^	12 (14.6)
**Infusion related complications** ^‡^	1 (1.2)
**Total adverse events grade 3–5** ^‡^	27 (32.9)

‡: n (%)

BV: brentuximab vedotin

A number of studies reported that administering BV in combination with other treatments, including various programmed death-1inhibitors, such as nivolumab and pembrolizumab, expanded the number of options for salvage therapies before and after AHSCT with and without relapse [5, 13, 15, 26, 27]. Along these lines, it has been reported that BV-based therapies had higher CR and ORR rates compared to other chemotherapy regimens [21]. On the other hand, a review reported that BV was a less cost-effective option than frontline or consolidation therapy [28]. We did not evaluate combination regimens in this study.

The use of BV before transplantation as a bridging therapy or after transplantation as a maintenance therapy has been debated in the literature [10]. Inconsistencies in the literature regarding response rates to transplantation and BV treatment may be attributed to the heterogeneity of patient groups. Husi et al. [3] reported that administering BV before transplantation did not have a positive effect on the OS and progression-free survival (PFS) of patients undergoing AHSCT. On the other hand, the AETHERA trial showed that administering BV as maintenance therapy after AHSCT improved PFS [29]. Due to the study’s retrospective design, we could not evaluate the efficacies of BV treatment practices that differed according to the timing of AHSCT.

The study’s retrospective design was its primary limitation. In addition, due to incomplete disease progression data, we could not analyze PFS. Another limitation of the study is the variability in the timing of reassessment scans across participating centers. While some institutions conducted reassessments at predefined intervals, such as after every three treatment cycles, others determined the timing based on clinical judgment or patient-specific factors. This variability may have influenced the consistency of response evaluation. However, this reflects the real-world nature of BV usage across diverse clinical practices and provides insights that are valuable for understanding its application in varied healthcare settings. On the other hand, the study’s primary strength was that it included real-world data obtained from 14 centers across Turkey. Additionally, gender equity was considered a significant factor in the design of this study. Our population comprises 56.1% female patients, indicating an attention to gender balance. During our analyses, potential effects of the gender factor, including examining gender-based responses, were taken into account. However, due to the retrospective design of the study, there are limitations in conducting detailed gender-based analyses.

The ORR of R/RcHL, patients to BV monotherapy, was 76.8%. In addition, the rate of patients with grade 3 to 5 toxicity to BV monotherapy was 32.9%, indicating a manageable side effect profile. In conclusion, BV monotherapy is an effective, reliable, and safe treatment modality in the treatment of R/RcHL patients, either as a bridging therapy to stem cell transplantation before AHSCT or alloHSCT or as a maintenance therapy after AHSCT or alloHSCT, with the latter being more effective. Prospective studies are needed to determine the specific characteristics of the subset of R/RcHL patients that will benefit from BV monotherapy.

## Conclusion

Brentuximab vedotin (BV) monotherapy demonstrates substantial efficacy and an acceptable safety profile in the treatment of refractory/relapsed classical Hodgkin’s lymphoma (R/RcHL) patients. This study highlights BV’s role as an effective bridging therapy to autologous and allogeneic hematopoietic stem cell transplantation (AHSCT/alloHSCT), which significantly improves overall survival (OS). Furthermore, neuropathy and hematological toxicities were observed as manageable adverse events, underscoring the importance of monitoring and supportive care during treatment. These real-world findings provide valuable insights into BV’s clinical use, supporting its integration into therapeutic strategies for R/RcHL. However, prospective studies are needed to further delineate the patient subsets most likely to benefit from BV and to establish its long-term efficacy and safety.

## Electronic Supplementary Material

Below is the link to the electronic supplementary material.

Efficacy and Safety of Brentuximab Vedotin Monotherapy in Refractory/Relapsed Classical Hodgkin’s Lymphoma: A Real-World Perspective
