# Supplementary material for: Outcomes of Brentuximab Vedotin Monotherapy in Refractory/Relapsed Classical Hodgkin’s Lymphoma: A Multi-Center Retrospective Study on Survival and Safety
Source: Indian J Hematol Blood Transfus. 2025 Jul 16;42(3):781–91. doi: 10.1007/s12288-025-02093-w (PMC13109441; doi:10.1007/s12288-025-02093-w)
Supplement: Supplementary file 1 — Efficacy and Safety of Brentuximab Vedotin Monotherapy in Refractory/Relapsed Classical Hodgkin’s Lymphoma: A Real-World Perspective [file 12288_2025_2093_MOESM1_ESM.docx]

Clinical Practice Point

Refractory/relapsed classical Hodgkin’s lymphoma (R/RcHL) has a poor prognosis and treatment response. The conventional treatment, autologous hematopoietic stem cell transplantation (AHSCT), leads to a survival advantage in most patients. An antibody-based salvage monotherapy called brentuximab vedotin (BV) was introduced to enhance efficacy. Studies have reported a better response and remission rate when combining AHSCT with BV and/or other medicines. Although there is promising evidence suggesting the success of BV monotherapy in cHL treatment, the adverse effects and toxicities associated with long-term BV use are poorly defined. Our findings describe the demographics and clinical factors related to patients and their disease progression. We found a notable overall treatment response rate of 76.8% and a median overall survival of 13.6 months, BV emerges as a vital therapeutic option. Patients who underwent HSCT following BV treatment demonstrated better survival, emphasizing the potential of BV as a supportive therapy for transplantation. Our study also describes the adverse effects that come with BV monotherapy. Although grade 3 to 5 adverse events were observed in approximately one-third of the patients, such as neutropenia, our findings demonstrated they are manageable, highlighting BV monotherapy's safety profile. The study findings highlight the importance of considering BV monotherapy as a key component in the treatment arsenal against R/RcHL, offering a promising treatment approach for R/RcHL patients.
